# Radiation-Induced Fibrosis in Head and Neck Cancer: Challenges and Future Therapeutic Strategies for Vocal Fold Treatments

**DOI:** 10.3390/cancers17071108

**Published:** 2025-03-26

**Authors:** Maria Jimenez-Socha, Gregory R. Dion, Camilo Mora-Navarro, Ziyu Wang, Michael W. Nolan, Donald O. Freytes

**Affiliations:** 1 Lampe Joint Department of Biomedical Engineering, North Carolina State University & University of North Carolina-Chapel Hill, Raleigh, NC 27606, USA; majimene@ncsu.edu (M.J.-S.); zwang77@ncsu.edu (Z.W.); 2 Comparative Medicine Institute, North Carolina State University, Raleigh, NC 27606, USA; mwnolan@ncsu.edu; 3 Department of Otolaryngology-Head & Neck Surgery, University of Cincinnati, Cincinnati, OH 45267, USA; diongy@ucmail.uc.edu; 4 Department of Chemical Engineering, University of Puerto Rico-Mayaguez, Mayagüez, PR 00680, USA; camilo.mora@upr.edu; 5 Department of Clinical Sciences, North Carolina State University College of Veterinary Medicine, Raleigh, NC 27606, USA

**Keywords:** head and neck cancer, radiation-induced fibrosis, voice disorders, treatments

## Abstract

Head and neck cancer encompasses a diverse group of malignant neoplasms originating in regions such as the oral cavity, oropharynx, hypopharynx, larynx, sinonasal cavities, and salivary glands. HNC represents a significant public health challenge, and recent reports indicate an increment in the incidence of HNC in young adults. In 2020, approximately 377,700 new HNC cases and 177,800 HNC-related deaths were reported globally. Major risk factors include tobacco smoking, alcohol consumption, and human papillomavirus (HPV) infections. HNC impacts vital functions such as breathing, swallowing, and speech. Treatments for this type of cancer within this complex anatomy include surgery, radiotherapy, and chemotherapy combinations. Radiotherapy is often an essential component of both curative and palliative HNC treatment, balancing tumor control with the preservation of function and appearance. However, its use can damage adjacent normal tissues, causing acute or chronic toxicity. One complication of HNC irradiation is VF fibrosis, which leads to severe voice impairments, significantly affecting patients’ quality of life. Fibrosis involves excessive and aberrant deposition of extracellular matrix, driven by factors such as TGF-β1 and inflammatory cytokines, which ultimately impair the flexibility and function of VF. Current radiation-induced fibrosis treatments primarily focus on symptom management and include systemic therapies like corticosteroids, anti-inflammatory drugs, and antioxidants. However, these treatments have limited efficacy. Experimental approaches targeting molecular pathways involved in fibrosis are being explored. Given the limitations of these treatments, advancing research is crucial to develop more effective therapeutic strategies that can significantly improve the quality of life for HNC patients, especially those vulnerable to VF fibrosis.

## Simple Summary

Head and neck cancer (HNC) is the seventh most common type of cancer and radiotherapy (RT) is a common treatment. One complication of HNC irradiation is fibrosis of the vocal folds (VF), which can adversely impact quality of life via alteration of voice. Here, we summarize what is known about the pathophysiologic mechanisms of radiation-induced fibrosis (RIF), and review clinical management strategies that have been developed to reduce fibrosis and restore tissue function of the VF. The overarching aim is to identify gaps in knowledge and windows of opportunity that could lead to new therapeutic options.

## 1. Introduction

**Figure 1:**
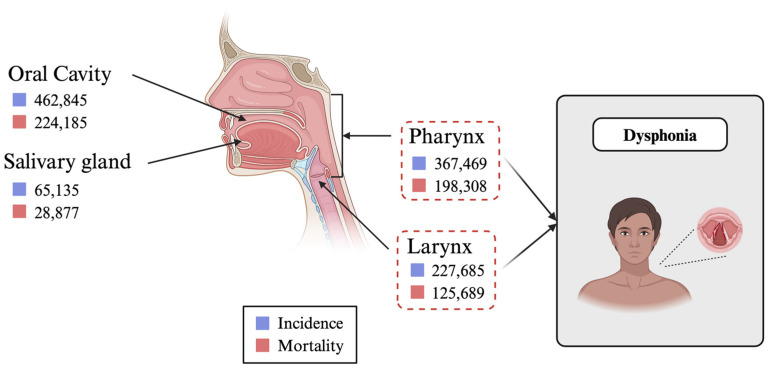
Projected incidence and mortality rates for various HNCs in 2030. The diagram highlights the prevalence of laryngeal cancers within the spectrum of head and neck malignancies and underscores their significant contribution to cases of dysphonia. Data reflect an upward trend in the burden of these cancers, emphasizing the need for targeted therapeutic and rehabilitative strategies to address voice-related outcomes [12]. Created with BioRender.com.

Head and neck cancers (HNC) comprise a diverse group of malignancies arising in the oral cavities, oropharynx, hypopharynx, larynx, sinonasal cavities, and salivary gland (Figure 1) [1,2]. HNC is a public health concern, accounting for approximately 4.5% of cancer diagnoses and deaths. It is considered the seventh most common cancer worldwide [2,3]. The risk of HNC is increased by factors such as tobacco smoking, alcohol consumption, and, more recently, human papillomavirus (HPV) infection. In the United States and Western Europe, the prevalence of tobacco-related HNC has decreased, whereas the incidence of HPV-associated HNC is increasing, especially in young patients [1,4]. In the 2000s, the overall incidence of HPV-positive HNCs in the USA has dramatically risen from 20% to 80% [4]. There is an expectation in early evidence suggesting that HPV vaccines will have a positive impact on reducing the affected population. Nonetheless, it is estimated that by 2030, the number of new cases will reach 1.08 million annually, making it a significant area of study [5,6].

While treatments such as radiotherapy (RT), chemotherapy, and surgery are being used for HNC patients to increase survival rates, these come with long-term side effects that can affect a patient’s quality of life. Patients with HNC have the highest rates of depression and suicide among all cancer types, which significantly impacts overall survival and quality of life [7,8,9]. In addition, long-term symptoms, such as dysphagia, pain, and voice problems, profoundly affect patients’ physical and emotional well-being, further diminishing their quality of life. A cross-sectional study of 1114 HNCs identified common long-term issues: xerostomia (46%), difficulty swallowing (40%), hoarseness (16%), and head/neck pain (14%), with only 5% reporting no issues. Logistic regression showed chemoradiotherapy increased the odds of xerostomia and dysphagia but decreased hoarseness and pain compared to surgery [10]. Advanced tumor stage and cancer type also influenced symptom prevalence. Based on an Eating Assessment Tool-10 (EAT-10) questionnaire, dysphagia significantly affects 54.9% of the HNC patients’ quality of life and strongly correlates with voice problems and pain [11].

Among all these symptoms, voice disorders are a common and debilitating complication, particularly in patients who have undergone treatment for HNC, especially in the larynx and pharynx. Voice disorders are characterized by abnormalities in pitch, loudness, or tone, with symptoms including hoarseness, vocal fatigue, and difficulty speaking [13]. A key contributor to these disorders is vocal fold (VF) fibrosis, a condition where scarring causes thickening and stiffening of the VF, impairing their vibration and leading to persistent voice issues [14,15]. This scarring, often a result of radiation, disrupts the natural function of the VF, resulting in hoarseness, reduced vocal range, and increased difficulties in speaking [13,16]. Understanding the link between VF fibrosis and voice disorders is crucial for developing targeted therapies to address these long-term side effects and improve the quality of life for survivors.

This review aims to summarize current knowledge on radiation-induced fibrosis (RIF) in HNC, with an emphasis on voice disorders. We will discuss the fibrotic response from radiation therapy, focusing on the underlying cellular and molecular mechanisms, such as fibroblast activation and extracellular matrix accumulation. Current treatments for managing RIF, including pharmacological interventions and non-pharmacological approaches, will be discussed. Specifically, we will address how radiation-induced fibrosis affects the VF, leading to voice disorders, and assess existing strategies to mitigate these effects. By analyzing current approaches, we aim to identify potential avenues for developing new therapies to address VF fibrosis resulting from radiation treatment.

### 1.1. Complexity and Treatment Modalities

About 40% of patients with locoregionally advanced HPV-negative disease eventually experience locoregional recurrence, which is a major contributor to the risk of death [17]. Moreover, approximately 10% of cases have confirmed distant metastatic disease at the time of diagnosis [2,6]. The frequency and significance of these late diagnoses and complex regional anatomy make HNC treatment challenging.

Combinations of surgery, RT, and chemotherapy are often used to treat HNC, and the exact approach is influenced by both the stage of the disease and the tumor site [18,19]. Even though effective HNC treatments exist, many patients will eventually experience either disease recurrence or progression or side effects of treatment [20,21]. Unfortunately, disease-induced or treatment-induced anatomical alterations can adversely impact breathing, swallowing, speech, smell, and vision [2,22].

The remainder of this review focuses on RT, the most widely used treatment for HNC. More than half of diagnosed HNC patients benefit from irradiation as a primary or adjunctive treatment [23]. Its role is pivotal in curative and palliative treatment, allowing tumor control and symptom relief [24]. The importance of RT in cancer treatment cannot be overstated because it balances tumor control and preservation of function and cosmetic appearances [25]. Still, the side effects of the therapy on the surrounding tissue remain a challenge, becoming a key aspect of this review of current strategies to promote tissue regeneration after treatment.

### 1.2. Mechanisms and Complications of Radiotherapy

RT is a commonly used treatment for patients with HNC, with conventional external beam RT being the most prevalent approach. Although traditional techniques may affect nearby healthy tissues, advances in RT have improved treatment accuracy [26,27]. Intensity-modulated radiation therapy (IMRT) and proton beam therapy are advanced techniques that have been developed to address some of the limitations of conventional RT. IMRT is designed to accurately treat complex-shaped tumors while reducing damage to surrounding healthy tissues. It creates highly conformal dose distributions using computerized inverse planning and multileaf collimators (MLC) [28,29]. On the other hand, proton beam therapy offers a more precise approach using Bragg peak, a phenomenon in which protons deposit most of their energy in a specific region of the tissue at the end of its trajectory. As a result, exposure to normal tissues is minimized while delivering lethal doses to the tumor and precisely covering the tumor volume [30,31].

**Figure 2:**
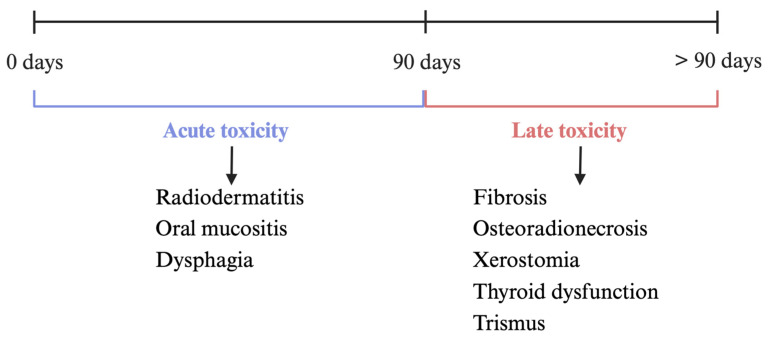
Visual representation of acute and late toxicity over time following radiation exposure in patients with HNC and examples of symptoms associated with each type of toxicity [19]. Created with BioRender.com.

While tumor cells tend to be more sensitive to ionizing radiation, injury can also occur in the nearby healthy tissues. Radiation toxicity is a major survivorship concern for HNC patients due to its impact on quality of life. Complications of RT can be divided into acute toxicity (occurring within 90 days) or late toxicity (>90 days post-irradiation) (Figure 2). Acute toxicities are typically temporary and self-limiting unless severe, while late toxicities are generally considered permanent. However, some acute toxicities can persist, and certain late toxicities may resolve over time [19,32,33].

Common acute effects in HNC patients are radiodermatitis and oral mucositis. Radiodermatitis presents much like a sunburn. Redness (erythema) and potentially dry desquamation are present in mild cases, along with itchiness. By contrast, severe radiodermatitis includes moist desquamation and can progress to ulceration. Patients can experience intense pain and/or burning sensations in these severe cases. There is no standard treatment for radiodermatitis, but basic hygiene and analgesic therapy are essential. Adjunctive treatments such as topical corticosteroids and hydrocolloid dressings may be prescribed based on clinician preference but without high-level evidence of efficacy [34,35]. In patients undergoing definitive RT, the incidence of acute oral or oropharyngeal mucositis approaches 90%. As with radiodermatitis, mucositis varies in severity, but patients with moderate to severe mucositis often have mucosal ulceration and pain that can limit their speech and ability to swallow. Treatments commonly used for mucositis include hydration, nutritional support, topical and systemically administered analgesics, and infection control as needed [36,37]. Patients with dysphagia (difficulty swallowing) may require nutritional supplementation via a temporary feeding tube; this intervention can help combat weight loss, malnutrition, and sarcopenia (loss of muscle mass) during RT [35,38]. An important consideration for HNC patients is the potentially additive toxicities that may occur as a result of combinatorial therapies. For example, Cooper et al. reported that 77% of patients receiving chemoradiotherapy had an acute effect compared to 34% who received RT alone [39].

Examples of the chronic (late) radiation effects most frequently encountered by HNC patients include osteoradionecrosis, xerostomia, thyroid dysfunction, and subcutaneous fibrosis. Osteoradionecrosis is a severe, delayed complication of RT, primarily caused by disruptions in metabolic and tissue homeostasis. It develops through a radiation-induced fibroatrophic mechanism, leading to progressive tissue damage, impaired healing, and exposure to irradiated bone [40]. Patients with osteoradionecrosis experience pain, bad breath, trismus, and speech difficulties. Management of osteoradionecrosis may include antibiotics, ultrasound, surgical resection, and reconstruction [41,42]. Xerostomia is caused by damage to the salivary glands and results in altered quantity and quality of saliva; these patients frequently experience constant pain. In patients undergoing definitive irradiation of the neck, the prevalence of xerostomia is between 73.5 and 93%, and some side effects include difficulties in eating, swallowing, and speaking [43]. Treatment options involve palliative use of salivary substitutes, non-pharmacological saliva stimulation, infection prevention, and drinking plenty of fluids [42,43]. Fibrosis can cause aesthetic and functional impairment. Patients with fibrosis may lose mobility in the neck, shoulders, and surrounding muscles, causing pain and trismus [42,44]. Some treatment options include physical therapy and topical and systemic treatments such as pravastatin, botulinum toxin, and myofascial release [42,45,46].

## 2. Radiation-Induced Fibrosis

Fibrosis is the excessive production and random deposition of extracellular matrix proteins, such as collagens as a disorganized reparative response to injury or damage. In patients undergoing RT for HNC, fibrosis can develop as a significant and often unavoidable complication due to the repetitive injury caused by the treatment [47].

**Figure 3:**
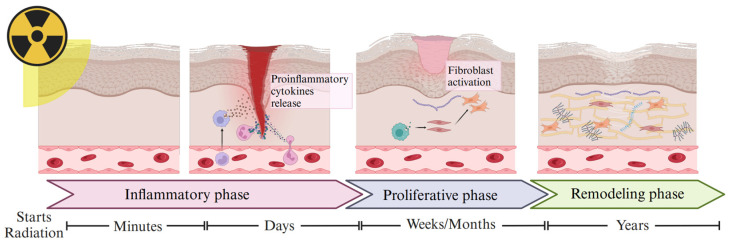
Mechanism of radiation-induced fibrosis. RT triggers a pathological process composed of three main phases: The inflammatory phase, characterized by cellular and tissue damage leading to the release of proinflammatory cytokines. This is followed by the proliferative phase, where fibroblasts are activated and differentiated into myofibroblasts. Finally, the remodeling phase, in which an imbalance in ECM homeostasis leads to its excessive accumulation and induces fibrosis. Created with BioRender.com.

The development of radiation-induced fibrosis involves various cellular and molecular pathways (Figure 3). Radiation exposure initiates a cascade of biological processes that lead to tissue damage and subsequent fibrotic remodeling. Analogous to tumor treatment, in the context of fibrosis due to radiation insult in the surrounding tissue, the radiation directly affects the nuclear and mitochondrial DNA structure, causing single-strand breaks, cross-links, and double-strand breaks (DSB). The DSB-related injury is the most common and complicated to repair while unrepaired DSBs contribute to efficacy in the tumor and toxicity in the normal tissues. Also, a secondary effect caused by radiation is generating reactive oxygen species (ROS) and reactive nitrogen species. The generation of ROS not only damages cells but also alters metabolism and epigenetic patterns that regulate how cells behave, contributing to the development of fibrosis and indirectly implicated in DNA damage [33,44,48,49,50].

Moreover, an inflammatory response is triggered during radiation-induced cellular damage, including the recruitment of immune cells to the affected site. Endothelial cells are primarily damaged and acquire a proinflammatory phenotype, increasing the expression of adhesion molecules such as VCAM-1, ICAM-1, and PECAM-1 [51]. These molecules facilitate the adhesion and recruitment of neutrophils from the bloodstream. Neutrophils are among the first inflammatory cells to reach the affected area and, upon contact with fragments of collagen and fibronectin, release proinflammatory cytokines such as TNF-a, IL-1, and IL-6, leading to exaggerated local inflammation and the onset of fibrosis [33,44,48,52,53,54].

Subsequently, monocytes and lymphocytes are incorporated into the process, in which monocytes differentiate into M1-like and M2-like macrophages. The author uses this nomenclature for simplicity, but macrophage polarization is complex and is out of the scope of the current review. In summary, M1 macrophages release chemokines and inflammatory cytokines such as IL-1β, IL-6, IL-12, IL-23, TNF-α, and ROS, all leading to the inflammatory response. M2 macrophages secrete growth factors that promote fibroblast migration to the damaged area, such as platelet-derived growth factor (PDGF) and growth factor. M2 macrophages also produce the TGF-β protein family, which promotes fibroblast proliferation as well as the key signaling protein family to promote myofibroblast phenotype and fibrosis [33,44,48,52].

TGF-β protein family is a key regulator of fibrogenesis and has five subfamilies, but TGF-β1 has been implicated in fibrosis. TGF-β can produce excessive deposition of extracellular matrix components because it drives fibroblast proliferation and activation. Once activated, fibroblasts transform into myofibroblasts, cells that play a crucial role in wound healing and fibrosis. This transformation is mediated by TGF-β signaling, promoting not only fibroblast proliferation but also recruitment to sites of fibrosis through its capacity as a chemoattractant. The process involves the activation of signaling pathways, the primary TGF-β/Smad pathway facilitating the translocation of Smad proteins to the nucleus to activate genes responsible for ECM production and tissue remodeling. In addition, other pathways promote fibroblast proliferation and migration and myofibroblast transformation, such as Rho/ROCK and PDGF/PDGFR. TGF-β positively regulates inhibitors of metalloproteinases (TIMP) and dysregulates the activity of matrix metalloproteinases (MMP-2 and MMP-9), resulting in an imbalance between TIMPs and MMPs, preventing degradation of both ECM and other extracellular proteins [33,44,48,52,55].

Therefore, excess ECM proteins, such as collagen, increase tissue stiffness, leading to tissue fibrosis over time [55]. In the VF, increased collagen production and deposition affect thyroarytenoid muscle remodeling and, in turn, the integrity of the muscle fibers, and ultimately leading to voice disorders [33].

Radiation-induced VF fibrosis and resulting dysphonia are estimated to occur in 10.5% to 64% of patients, depending on the treatments performed and the duration of follow-up [56]. The median time to onset of radiation-induced vocal cord fibrosis and dysphonia usually begins about 4 to 6 weeks after RT. Typically, the severity of dysphonia usually peaks at about 10 weeks post-radiation, where, in most cases, the voice never returns to normal (pre-treatment) levels. With standard radiation fractionation, patients at greatest risk of dysphonia are those whose laryngeal dose is greater than 50 Gy [56,57,58].

Several factors influence the progression and severity of radiation fibrosis. Age is crucial; as people age, physiological changes affect normal tissue function and decrease tolerance to RT. Physiological changes such as deterioration of tissue structure and functionality and a decrease in the compensatory capacity of muscles cause older people to be impaired in speech [59,60]. In addition, vulnerability to side effects also increases with age, where patients over 65 years of age have a toxicity rate of 56% versus 31% for patients under 50 years of age [61,62].

Additionally, tumor size and radiation dose also contribute to the risk and severity of fibrosis in patients with HNC. Patients often present with speech dysfunction at doses greater than 50 Gy to the lateral pharyngeal wall and false VF. Since radiation affects the tumor and normal tissues, it alters their architecture and mobility and causes voice alterations [59,63].

Genetics have also been shown to influence the development of fibrosis. For example, ataxia telangiectasia mutated (ATM) is responsible for repairing DNA double-strand breaks; however, when a gene mutation alters the repair mechanism, the tissue response to radiation is also altered, leading to fibrosis. Radiosensitivity increases at low doses and has been related to the presence of the G→A polymorphism in nucleotide 5557 of the ATM gene compared to unaltered patients [64,65].

### 2.1. Current and Experimental Treatments for Radiation-Induced Fibrosis

The treatment of radiation-induced fibrosis in patients with HNC remains a challenge. Currently, no treatments help reverse radiation-induced fibrosis. However, some approaches, which may be systemic, topical, or mechanical, primarily aim to manage symptoms and improve functional outcomes.

#### 2.1.1. Systemic Therapies

Systemically administered corticosteroids and non-steroidal anti-inflammatory drugs regulate the TNF-α and TGF-β1 pathways involved in fibrosis [15]. Cytokines associated with inflammatory pathways are drivers of fibroblast activation and fibrosis in the long term. Pentoxifylline can reduce inflammation by inhibiting TNF-α, and its rheological effects improve microcirculation and oxygenation [66]. It has been combined with vitamin E, which protects membrane phospholipids from oxidative damage. The combination of pentoxifylline and vitamin E has shown promise in reducing fibrosis either singly or combined due to inhibiting intracellular signaling in response to the TGF-β signaling pathway [44,67]. However, there is little evidence that it reverses the side effects of radiation and is associated with systemic side effects.

Lycopene is an effective antioxidant obtained from tomatoes. The mechanism of action is that it is a radical scavenger and singlet oxygen suppressor. It has shown promising results as a drug with both anti-inflammatory and antioxidant properties [68]. Additionally, therapeutic ultrasound combined with soft tissue mobilization and lycopene injection was explored in managing patients with oral submucous fibrosis, which offers a synergistic effect, helping to alleviate symptoms and improve tissue flexibility [69].

Imatinib, a clinically available tyrosine kinase inhibitor, has demonstrated efficacy in reducing PDGFR-β phosphorylation and TGF-β expression, key mediators of the fibrotic process [70]. Imatinib has been used in radiation-induced dermal fibrosis because this fibrosis results from the overactivation of PDGFR-β and TGF-β signaling, driving excessive collagen deposition and tissue thickening [71]. Pravastatin is a phase II drug used to treat HNC patients with cutaneous and subcutaneous fibrosis. It has antifibrotic potential because it reduces the effects of radiation-induced fibrosis in patients. It could be considered a secondary treatment for patients with severe cutaneous RIF but needs to be confirmed with phase 3 trials [46]. Nintedanib, an FDA-approved tyrosine kinase inhibitor, reduces fibrosis by inhibiting key pathways involved in fibroblast activation, including TGF-β/Smad, PI3K/AKT/mTOR, and MAPK. It prevents the fibroblast-to-myofibroblast transition by downregulating fibrotic markers such as α-SMA, COL1A1, and CTGF and suppresses inflammatory cytokines like IL-6 and IL-1β [72]. Early administration of Nintedanib is particularly effective in mitigating fibrosis, which indicates its potential for preventing fibrotic progression and inhibiting the development of scar tissue. Table 1 summarizes the systemic therapies currently being studied for treating fibrosis.

Due to the small size and highly localized nature of the VF area, the systemic administration of molecularly targeted therapies (Table 1) presents significant challenges in achieving therapeutic concentrations at the target site. High doses would be required to ensure adequate drug delivery to this micro-targeted region, resulting in an increased risk of systemic toxicity. To tackle this limitation, various localized drug delivery strategies have been investigated to enhance therapeutic efficacy while minimizing the potential for adverse effects. One such approach, intra-arterial chemotherapy, has evolved significantly from its initial application in treating liver malignancies to become a widely explored modality for addressing a diverse range of cancers. This strategy aims to reduce systemic side effects and improve the pharmacological effectiveness of chemotherapeutic agents by delivering them directly to the tumor site. Administration via the laryngeal artery has been investigated as a method to achieve higher local drug concentrations while minimizing systemic exposure, demonstrating efficacy in the treatment of HNC [73]. Clinical trials, such as those by Rao et al. [74] and Yu et al. [75], indicate promising results in patients with locally advanced HNSCC, with survival rates and long-term disease-free outcomes. Furthermore, super selective intra-arterial chemoradiotherapy, which combines localized delivery of chemotherapeutic agents with radiation therapy, has been employed to target specific cancers, including those of the maxillary sinus [76]. This dual modality has shown promise in maximizing treatment effectiveness while minimizing damage to surrounding healthy tissues.

#### 2.1.2. Topical Treatments

Topical treatments, including gels and creams containing anti-fibrotic agents, target localized areas of fibrosis, especially in cases where skin and superficial tissues are affected. Superoxide dismutase, which is used topically, is an antioxidant enzyme that acts as an endogenous defense against oxygen radicals. Although oxidative stress is one of the pathophysiological mechanisms for radiation damage, no relevant results have been shown when treating post-radiation fibrosis with superoxide dismutase cream [44,64,77].

#### 2.1.3. Mechanical Treatments

Mechanical treatments like physiotherapy and speech therapy play a crucial role in managing the functional aspects of fibrosis, particularly in maintaining mobility and function in the affected areas. In addition, physical therapy improves skin perfusion and vascularization, which may partially reverse radiation-induced fibrosis. Movement-based rehabilitation exercises, such as resistance training (RET) combined with joint mobility exercises and stretching, have reduced pain and improved function in patients receiving RT [78].

However, treating RIF presents significant challenges. First, current therapeutic options are limited in efficacy, with many patients experiencing only partial relief of symptoms. Second, although attempts have been made to improve radiation techniques, toxicity still occurs when using this treatment. This results in the formation of acute tissue wounds that subsequently develop into fibrosis and may continue to evolve until after the end of treatment. Moreover, the heterogeneity of patients’ responses to treatment underscores the need for personalized therapeutic approaches.

#### 2.1.4. Experimental Treatments

New avenues for treating radiation-induced fibrosis, including new pharmaceutical agents targeting specific molecular pathways in fibrosis development, gene therapy approaches, and stem cell therapies, are being explored. The effectiveness of these emerging treatments is still under investigation, with the hope that they will offer more targeted and effective solutions for managing fibrosis and improving the quality of life for HNC patients.

TGF-β1 plays a key role in fibrosis by modulating fibroblast function. Therefore, TGF-β1 pathway modulation becomes a potential target for treating fibrosis in HNC patients. TGF-β1 receptor kinase inhibitors, such as galunisertib (LY2157299), vactosertib (EW-7197), LY2109761, SKI2162, and fresolimumab (GC1008), reduce inflammation, fibrosis, and matrix deposition by blocking TGF-β1 signaling [54,64,79]. However, the development of TGF-β1 as a clinical intervention faces some concerns because, although it is a mediator of fibrosis, it also has essential functions in processes such as hematopoietic cell differentiation and regulation of the immune system. Therefore, its inhibition could trigger adverse effects such as autoimmune diseases, making it a challenge to selectively block the profibrotic actions of TGF-β1 without causing harm to the patient [80]. PDGF is another potential target for treating fibrosis because it induces the differentiation of the fibroblasts into myofibroblasts and the production of collagen. Therefore, it would reduce excess scar tissue and decrease fibrosis using PDGF inhibitors such as SU9518 and SU14816 (imatinib). However, this target has only been studied for radiation-induced pulmonary fibrosis (RIPF). Therefore, the implications for fibrosis generated in the head and neck could be investigated [81,82].

Another field of interest is the application of advanced biotechnologies, such as stem cell therapy. For example, adipose tissue-derived stem cells (ASCs) can suppress TGF-β1 expression and thus aid in treating fibrosis [83]. In addition, ASCs have immunomodulatory capacity by reducing the production of proinflammatory cytokines, resulting in decreased inflammation of damaged tissue and subsequent fibrosis [84].

Extracellular vesicles (EVs) were initially classified as a cell waste management mechanism, but nowadays, they are part of cell communication and possess a biomolecular cargo capable of modulating cell fate response in either surrounded tissue or in system applications. EVs are harnessed from cell culture media and represent an acellular potential treatment option that has been shown to mitigate the adverse effects caused by radiation on different tissues and organs. EVs derived from bone marrow mesenchymal stem cells (BMMSCs) showed in vitro and in vivo improvements in bone marrow recovery from irradiation. BMMSC EVs mitigated adverse effects by promoting cell proliferation and reducing apoptosis, facilitating hematopoietic regeneration. Furthermore, it has been found that EVs derived from umbilical cord MSCs in lung injury caused by irradiation can inhibit fibroblast differentiation into myofibroblasts, an important process involved in fibrosis [85].

In addition, advances in radiation techniques, such as intensity-modulated radiation therapy (IMRT), in which high doses are delivered to the tumor while preserving the integrity of normal tissue, reduce radiation toxicity. Also, proton therapy aims to reduce the degree of exposure of healthy tissues to radiation by delivering precise radiation doses that are adjusted to the shape of the tumor from multiple angles, which could reduce treatment toxicity such as fibrosis [86,87,88].

Decellularized ECM scaffolds (dECM) are bioinstructive scaffolds in which the cellular components of the ECM are removed and resemble the native tissue environment; studies have shown that dECM is a good option in functional tissue regeneration because it promotes tissue healing [89,90]. In addition, it has been considered a promising treatment for cancer patients, where dECMs are used in reconstructive surgeries after tumor removal [91]. For example, dECM has been used after surgery in patients with intramucosal adenocarcinoma, and improvement in patients has been observed due to the ability to modulate the innate immune response and attract endogenous stem cells, allowing esophageal regeneration [92]. It has also been studied in patients with oral and oropharyngeal defects, where a dECM graft was used for tissue integration and healing due to stimulation of cell adhesion, proliferation, and migration [93].

Although dECM has not been studied in patients with post-radiation alterations, its ability to regenerate and restore damaged tissue shows potential in HNC patients. Since radiation damages the tissues around the tumor, the regenerative properties of dECM could facilitate effective healing and reconstruction, minimizing fibrosis as a potential outcome. Table 2 provides an overview of the experimental treatments currently under investigation for fibrosis.

In the treatment of radiation-induced fibrosis, various approaches are being explored to reduce or reverse fibrosis. However, the above treatments have only been used in clinical trials and are not currently used as a routine clinical treatment for radiation-induced fibrosis. Current treatments for HNC patients that address radiation side effects include amifostine, the only FDA-approved drug for the treatment of xerostomia in HNC patients, which has been associated with improvements in radiation-induced oral dryness. However, its use for radiation-induced fibrosis is off-label. Amifostine, a radioprotective compound for HNC. It is effective but toxic when used at high doses, so clinical use must be controlled. Some of the side effects that patients have presented are a decrease in blood pressure, nausea, and a narrow therapeutic, for which reason it has had to be discontinued in some patients. The efficacy of amifostine is ascribed to free-radical scavenging, along with DNA protection and repair. This drug is designed to reduce the incidence of moderate to severe xerostomia in patients, so a treatment for fibrosis has yet to be discovered [97,98,99].

To prevent radiation-induced fibrosis, the risk factors of radiation dose and volume of irradiated tissue must be considered initially. Although new technologies have attempted to minimize these potential factors, side effects are still common with this treatment [54,64]. Therefore, the mechanisms of fibrosis and possible targets for developing strategies to help treat fibrosis should be further investigated.

## 3. VF Fibrosis and Treatment

**Figure 4:**
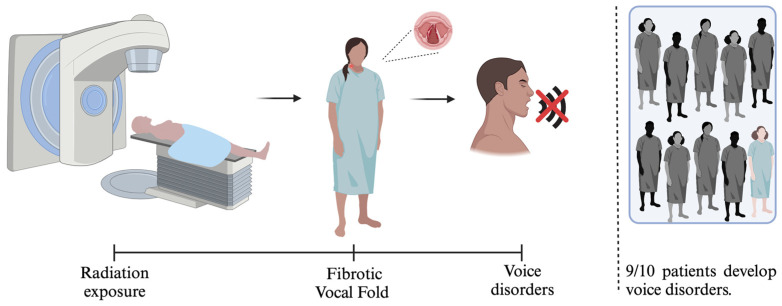
Schematic of the pathologic process in patients with HNC undergoing RT. It shows the induction of fibrosis in tissues by RT, which results in structural and functional alterations associated with voice disorders. Approximately 90% of patients treated with radiation develop voice disorders, evidencing the high prevalence of this side effect [101]. Created with BioRender.com.

VF fibrosis is one of the special cases of radiation-induced fibrosis. It causes dysphonia due to VF damage, affecting its ability to vibrate and, therefore, voice production [100]. Whenever an RT field includes the larynx, there is a risk for radiation-induced VF fibrosis [101]. As the mucosal layers of the VF become fibrotic, there is a progressive loss of tissue pliability, vocal cord asymmetry, and reduced range of motion (Figure 4) [33,100,102]. This can lead to vocal impairments ranging from mild dysphonia to significant voice loss [19,101].

This tissue injury impedes the patient’s ability to communicate and significantly affects the quality of life [15,33]. It has been estimated that more than 87% of patients perceive their voice as abnormal after laryngeal irradiation [101]. However, current treatment options for post-RT dysphonia are limited, and there is no established protocol to address this problem. As a result, patients suffer from chronic dysphonia without access to effective treatment to restore their voice. Regenerative medicine and tissue engineering offer promising strategies to restore VF function after radiation therapy-induced fibrosis. Utilizing stem cells and bioengineered scaffolds that mimic the native VF tissue makes it possible to help regenerate healthy tissue and reduce scarring. These approaches aim to reverse the stiffening of the VF caused by fibrosis, thereby improving their elasticity and vibratory capacity, which is essential for voice production. Implementing such advanced therapies can significantly enhance the quality of life for patients by restoring their vocal abilities and reducing the long-term side effects of radiation treatment.

Some treatments used for RIF, such as pentoxifylline with vitamin E, lycopene, imatinib, pravastatin, and nintedanib, have been studied in other organs and tissues but have not been explored in VF fibrosis. All these substances have anti-inflammatory properties, making them potential treatments for VF fibrosis because some modulate signaling pathways associated with cell damage and fibrosis, such as those mediated by TGF-β and PDGF receptors. Based on its key targets, it would be beneficial to include it in future research for treating radiation-induced VF fibrosis.

### 3.1. Traditional VF Treatments

Injection laryngoplasty is one of the most common treatments for scarred VF. The injected solution is delivered to help collagen synthesis and/or regulation of TGF-β1 signaling, improving voice quality. Currently, anti-inflammatory medicines such as steroids and dexamethasone are routinely used to alter the amount of collagen deposition by dampening the inflammatory response [15]. Hyaluronic acid (HA) has become common in treatment due to its optimal viscoelastic properties and its role in the maturation and maintenance of the lamina propria of the VF. However, the effects are not permanent because the materials used with this treatment have not shown the ability to completely restore the altered lamina propria, allowing it to return to its pre-irradiation vocal [15,103,104].

Additionally, synthetic polymers such as polyethylene glycol (PEG) are used as bulking agents in VF treatment to enhance phonation. PEG-based hydrogels are biocompatible and can be injected into scarred VF, where they provide temporary volume augmentation, improve glottal closure, and facilitate better VF vibration during speech. These synthetic polymers fill the extracellular space within the damaged tissue, reducing the gap caused by fibrosis and allowing for more efficient sound production. As the PEG hydrogel slowly degrades, it may also support tissue remodeling and encourage the regeneration of healthier VF tissue [105,106,107]. This approach complements regenerative medicine strategies by providing immediate functional improvements while longer-term tissue healing processes occur.

However, these treatments do not reverse the scar formation or help regenerate towards a functional lamina propria. They remain short-term strategies to help provide some relief to the patients.

### 3.2. Novel VF Treatments

Given the limited therapeutic options currently available for restoring VF function, new approaches that leverage knowledge from treating fibrosis in other tissues are being investigated. By applying insights from current fibrosis research and regenerative medicine, these novel strategies aim to provide practical solutions to reverse damage and restore the delicate structure and function of the VF.

The potassium titanyl phosphate (KTP) laser has been shown to improve VF flexibility and vibration. However, its efficacy is influenced by scar thickness and the affected area. The significant improvement presented in treatments with angiolytic lasers is their ability to negatively regulate the TGF-β pathway and reduce inflammatory mediators such as IL-1β, COX-2, and TNF-α. Therefore, this treatment contributes to restoring the damaged structure of the VF [108,109].

Pirfenidone, an FDA-approved drug for treating idiopathic pulmonary fibrosis, is an antifibrotic agent with anti-inflammatory and antioxidant properties. Its mechanism regulates gene expression signaling from pro-fibrotic cytokines [110]. However, it has been used to study the effect of pirfenidone injection on VF scars in an in vivo study. For example, Yamada et al. reported that using pirfenidone injections to treat scarred VF reduces type I collagen and α-SMA levels, resulting in less myofibroblast activation. Although pirfenidone seems a promising option for treating VF scars, the half-life of this drug in its standard form is less than one hour, making the treatment insufficient. While the study by Yamada et al. used sustained release (Kollidon SR) to address the problem, no information on prolonged bioavailability was presented. Therefore, long-term studies should be conducted using pirfenidone injections to determine its potential in treating VF scars [111].

Mitomycin-C (MMC) is an antibiotic that has shown antiproliferative activity by inhibiting fibroblasts. It has been studied as an adjuvant treatment for inhibiting fibrosis and scar formation. Random clinical trials have shown that adding MMC with RT in treating HNC improves local control and disease-free survival without increasing normal tissue toxicity [112,113]. In vitro and in vivo models have studied the antifibrotic capacity of MMC in VF. As a result, MMC reduces the expression of markers such as α-SMA, making it a potential treatment to reduce VF fibrosis [114,115].

Stem cell therapy has been investigated for VF restoration, where the cells primarily used are bone marrow-derived MSCs and ASCs. Stem cell therapy has shown positive results in reducing the stiffness of the VF and improving its mechanical properties [116,117,118]. For example, Svistushkin et al. demonstrated the potential of MSC-based therapies using a PEG-fibrin hydrogel composite and human bone marrow-derived MSCs to enhance VF regeneration after chronic damage. The results obtained were that MSCs showed significant anti-inflammatory capacity, and their paracrine mechanism stimulated epithelial repair and improved tissue quality. Also, using the hydrogel made it possible to maintain cell retention at the implantation site for more days than when the cells do not have a carrier, thus favoring healing [118].

The ongoing landscape of treatments for voice disorders in HNC patients treated with RT reveals a crucial gap in effective solutions, in particular to address fibrosis-induced VF damage. The focus is on innovative therapies to regenerate damaged tissue and reduce fibrosis. The emerging idea for treating VF fibrosis in HNC patients after RT is to utilize the properties of a decellularized porcine VF lamina propria extracellular matrix (VFLP-ECM). VFLP-ECM has demonstrated promising antifibrotic effects by modulating the TGF-β1 pathway, which is essential in fibroblast activation and scar formation [96]. This work represents a novel approach to providing a functional and structural solution to RT-induced VF fibrosis, allowing patients to preserve their voice and improve their quality of life after treatment.

## 4. Conclusions

RT is a cornerstone of HNC treatment and provides a route for controlling tumors while preserving cosmetic appearance. Despite its efficacy, the side effects of RT can be severe, and a notable example is VF fibrosis, which can be debilitating. The pathogenesis of VF fibrosis is multifaceted. It involves dysregulation of the normal wound healing processes, primarily driven by upregulation of TGF-β and myriad inflammatory cytokines. These molecular changes lead to excessive collagen deposition, resulting in tissue stiffening and normal VF function loss.

Various pharmacological agents have been investigated to prevent or mitigate VF fibrosis, targeting different aspects of the underlying molecular pathways. However, these efforts have yet to produce a consistently successful clinical intervention. This lack of effective treatment options highlights the urgent need for ongoing research into the mechanisms driving fibrosis and the development of innovative therapeutic strategies. Addressing this challenge is essential for improving the quality of life in HNC patients undergoing RT who are at risk or currently battling VF fibrosis.

## Figures and Tables

**Table 1:** Summary of systemic therapies for treating radiation-induced fibrosis.

Systemic Therapies	Mechanisms	References
Pentoxifylline with vitamin E	Inhibition of intracellular signaling in response to TGF-*β* (family of protein)	[44,67]
Lycopene	Radical scavenger	[68]
Imatinib	Inhibit PDGF receptor	[70]
Pravastatin	Antifibrotic potential	[46]
Nintedanib	Inhibit pathways suck as TGF-β/Smad, PI3K/AKT/mTOR, and MAPK	[72]

**Table 2:** Summary of experimental treatments for treating radiation-induced fibrosis.

Experimental Treatments	Examples	Mechanisms	References
TGF-β1 receptor kinase inhibitors	LY2157299, EW-7197, LY2109761, SKI2162, and GC1008	Reduce inflammation and fibrosis by blocking TGF-β1 signaling	[54,64,79]
PDGF inhibitors	SU9518 and SU14816	Reduce excess scar tissue	[81,82]
Stem cell therapy	Adipose tissue-derived stem cells	Suppress TGF-β1 expression	[84]
Advances in radiation techniques	Intensity-modulated radiation therapy	High doses are delivered to the tumor site	[86,87]
Extracellular vesicles (EVs)	EVs derived from bone marrow mesenchymal stem cells (MSCs)	Promote cell proliferation and reduce apoptosis	[85]
	EVs derived from primed cells, such as interferon-gamma MSCs or fibroblasts	EVs isolated from primed cells have been shown to modulate the TGF-β1 pathway through their molecular cargos	[94,95]
	Matrix-bound vesicles (BVs)	MBVs and macromolecules isolated from the VF lamina propria ECM have been reported to inhibit alpha-smooth muscle actin in fibroblasts stimulated through TGF-β1	[96]
Decellularized ECM scaffolds	Use in intramucosal adenocarcinoma, oral and oropharyngeal defects	Promotes tissue healing	[92,93]
